# Which Metrics Are Appropriate to Describe the Value of New Cancer Therapies?

**DOI:** 10.1155/2015/865101

**Published:** 2015-06-16

**Authors:** Peter Johnson, Wolfgang Greiner, Imad Al-Dakkak, Samuel Wagner

**Affiliations:** ^1^Cancer Research UK Centre, University of Southampton, Southampton SO16 6YD, UK; ^2^Department for Health Economics, University of Bielefeld, 33501 Bielefeld, Germany; ^3^PAREXEL International, 160 Euston Road, London NW1 2DX, UK; ^4^Bristol-Myers Squibb, Route 206 and Province Line Road, Princeton, NJ 08543, USA

## Abstract

Patients with certain cancers are treated with curative intent, but for others the results are less favorable and different therapeutic approaches are needed. Early data suggest that new therapies, which modulate immune responses to cancers, may have potential for long-term survival in a proportion of cases. Therefore, it is timely to consider whether metrics generally used to describe the medical value of therapies for patients with common solid tumors remain appropriate for therapies with curative potential. Literature reviews were conducted to define how various stakeholders describe cure in oncology and to identify the endpoints used in clinical trials for selected solid tumors. The results showed that “cure” is described using various terms that can be divided broadly into lack of disease progression, eradication of cancerous cells, and survival. The review of trial endpoints showed frequent use of median overall survival (OS) and progression- and response-related endpoints. Because these endpoints were mainly described in the context of chemotherapies that are not generally curative, they may not adequately capture outcomes of new therapeutic modalities with potential for long-term survival. More appropriate endpoints may include mean OS, cure fraction, and OS rate at landmark time points.

## 1. Introduction

The intentions and expectations of cancer therapy differ substantially depending on the type of malignancy and its extent. There are patients with cancer whose treatment can be curative, meaning that these patients no longer have active malignancy and will eventually die of something other than the cancer. In these cases, despite the different biology of the cancers and their treatment, what “cure” actually means is similar for all. Examples of curative cancer therapies include the surgical resection or radical irradiation of early epithelial cancers; adjuvant chemotherapy for breast and colon cancer; systemic therapy for childhood tumors such as acute lymphoblastic leukemia, osteosarcoma, or neuroblastoma; and systemic therapies in adults for germ cell tumors, some lymphomas, and acute leukemias. However, for most patients with advanced solid tumors, treatment is not given with curative intent, rather with the expectation of improving survival by some time and/or alleviating symptoms. In a review of new drug approvals by the United States Food and Drug Administration (FDA) between July 2005 and December 2007, approval was based on an effect on overall survival (OS) for only 10 of the 53 agents approved [1]. Consequently, the description and assessment of cure have not been central to the reporting of results in the treatment of these patients [2]. The generally poor prognosis for patients with most types of advanced solid tumors continues to drive research to identify and develop new therapeutic approaches that offer the potential for long-term survival. The ultimate goal is to achieve a durable survival benefit that allows patients to be considered “cured.”

The concept that autologous immune surveillance fails during the development of cancer and that the restoration of this recognition might produce therapeutic benefit has long been investigated [4]. The possible feasibility of this in practice has been recognized with the recent development of active immunotherapies designed to modulate the patient's immune system directly, thereby overcoming the mechanisms by which tumors evade recognition and elimination. Examples include ipilimumab, a cytotoxic T-lymphocyte antigen-4 [CTLA-4] immune checkpoint inhibitor, as well as nivolumab and pembrolizumab, programmed death-1 [PD-1] immune checkpoint inhibitors [5, 6]. These forms of treatment appear to act quite differently to conventional cytotoxic drugs or small molecule enzyme inhibitors, in that a proportion of the patients treated derive long-term benefit with prolonged suppression of the malignancy and no evidence of regrowth despite several years of follow-up [3, 7–10]. This is in-keeping with the known mechanisms of immunity, which generally confers almost life-long protection in the case of infectious diseases.

The recognition that there may be new modalities of cancer treatment, immunotherapy in particular, with the potential for long-term survival and possibly cure warrants a review of whether the metrics normally used to evaluate new compounds for advanced solid tumors are appropriate and accurately capture the important treatment outcomes [2, 5]. Against this background, it is helpful to consider how “cure” is currently described and assessed for patients with cancer.

## 2. Terms Used to Describe Curative Cancer Treatments

A review of literature from four sources (journal articles, conference and congress proceedings, health technology assessment [HTA] reports, and selected websites) published in English between January 1, 2007, and November 14, 2012, was conducted to identify “cure” definitions. To verify the appropriate choice and use of search terms, the search was validated against two relevant articles [11, 12]. Additionally, it was evaluated whether the definition varied according to the stakeholder or group describing it (i.e., academics, healthcare professionals, patients and patient interest groups, or payers). The literature search strategy was designed pragmatically to prioritize the identification of key information in a very large literature base. Notably, the review did not identify any published reports exploring the definition of cure broadly across oncology. Please refer to Supplementary Material 1 for detailed search methodology, limitations, outcomes, and results (see Supplementary Material available online at http://dx.doi.org/10.1155/2015/865101).

The literature review identified 3,932 documents, of which 169 were included following screening. The final sample included journal articles (*n* = 83), HTA documents (*n* = 7), conference abstracts (*n* = 56), and documents from patients/patient advocacy organizations (*n* = 18) and health care professionals' (*n* = 5) websites. Briefly, the results of the literature review showed that, across the materials evaluated, cure could be described using one or more of the following broad categories, with different descriptions or metrics used for assessment within each category:

1. *lack of disease progression*, including complete remission, disease-/event-/recurrence-/progression-free survival, functional cure, and time to treatment failure,
2. *eradication of cancerous cells*, including terms related to the removal of cancerous cells (by various therapeutic modalities, radiation therapy, and killing of cancer cells),
3. *survival*, including landmark OS rates (from 2 to 20 years), median OS, cure models, cure fraction, the patient dying from noncancer-related causes, and the mortality rate aligning with the general population; in this category, most groups discussed cure in the context of a follow-up period of about 5 years.

The results from this review revealed differences between groups' opinions on what describes a cancer “cure.” Healthcare professionals and academics placed a larger emphasis on disease progression and survival, while patients and payers focused on the successful eradication of cancerous cells. The percentage of documents using survival, lack of disease progression, or lack of disease progression plus survival as cure definitions was 35%, 28%, and 20%, respectively, for healthcare professionals; 39%, 39%, and 0%, respectively, for academics; 22%, 0%, and 0%, respectively, for patients/patient advocacy organizations; and 0%, 43%, and 0%, respectively, for HTA agencies (see Supplementary Figure 2).

Moreover, many of the descriptions, particularly as used by clinicians, were associated with cure in the context of hematologic malignancies. Of the 60 documents identified for healthcare professionals that contained cure definitions, 43 (72%) were in relation to hematologic malignancies compared with 15 (25%) for solid tumors (two reports had generalized content). This is not surprising since relatively a few therapies for solid tumors offer the potential for long-term survival, while, for certain hematologic malignancies, treatments are given with curative intent [13, 14]. As reflected in the results, cure can be described in terms of survival, measured as a proportion of patients who die of causes other than their disease if the follow-up period is long enough, or as a surrogate such as the proportion of patients alive at a time point appropriate to the type of cancer (e.g., 5 years for rapidly recurring cancers or 10 to 20 years for those that typically relapse later). Another component to the description of cure is more functional, relating to the time during which patients remain free from cancer progression or the effects of cancer on their health.

The use of descriptions relating to the eradication of cancer cells, particularly in documents from patients and payers, is thought provoking (please refer to Supplementary Material 1, Table  1, and Figure  2). It can never be proven that all the tumor cells have been destroyed, but rather they simply do not manifest themselves, allowing the patient to live without overt cancer until death from another cause (therefore, they are “cured” of the original disease). It can be proposed that treatments that are given for a relatively short duration and result in a long-term survival with no evidence of tumor recurrence, such as surgical resection for early epithelial cancer or adjuvant therapy for breast cancer or ipilimumab for metastatic melanoma, may completely eradicate the tumor, although this is not fully provable. In some hematologic malignancies, immunophenotypic or molecular techniques show that malignant cells persist in the absence of clinical signs or symptoms (termed minimal residual disease (MRD)), meaning that there is potential for tumor regrowth. For chronic myeloid leukemia, continued therapy with agents such as imatinib or dasatinib may be needed to maintain tumor control while treatment continues, despite the presence of such MRD [15]. In these settings, use of the terms operational or functional cure has become common [16]. In this context, describing cure as the eradication of cancer cells is neither measurable nor accurate. Although the results suggest that this is how certain groups describe cure, in clinical practice, curative cancer therapies may not align with that definition.

Understanding that cure is not always described or assessed in the same way and is not frequently used for advanced solid tumors since relatively a few therapies are given with curative intent, a further literature search was conducted to identify which endpoints are commonly used to assess treatments for patients with malignant melanoma, non-small cell lung cancer (NSCLC), and renal cell carcinoma (RCC) in clinical trial settings. This information was then used as a basis to address whether the commonly used endpoints would be appropriate to assess the medical value of therapies with the potential for long-term survival and how/whether these endpoints may support the notion of a “cure” in the way normally understood.

## 3. Endpoints Used to Assess Treatment Outcomes in Malignant Melanoma, NSCLC, and RCC

A literature review of information published in English between January 1, 2007, and December 31, 2012, was conducted to identify the clinical endpoints used in malignant melanoma, NSCLC, and RCC clinical trials and their frequency of use. These tumors were selected as they are the main types in which novel immunotherapies have been evaluated to date. Only endpoints directly referring to clinically measured outcomes were included, and not point estimates such as hazard ratio (HR) which are derived from data analysis. Two literature sources were searched: published systematic reviews of clinical trials and HTA reports. Additionally, documents identified during the first search that also provided metrics (in conjunction with cure definitions) were included in the results of this second search. Full details of the methodology and limitations are included in Supplementary Material 2. Again, this literature search strategy was designed pragmatically to prioritize the identification of key information in a very large literature base.

The literature search identified 2,951 documents, of which 146 were included in the final sample, comprising 92 clinical trial review papers and 54 HTA documents. The endpoints identified in the clinical trial reviews and HTA reports are shown in Tables 1 and 2, respectively. Overall, the most common endpoints reported were response rate, disease-free survival (DFS) or progression-free survival (PFS), median OS, recurrence rate, and quality of life. The endpoints used to assess clinical response were the most heterogeneous and, within this category, response rate or objective response rate (ORR) was the most frequently used. These endpoints were also used across all evaluated tumor types. Endpoints related to disease progression were relatively homogeneous, with PFS, DFS, recurrence-free survival, and time to progression (TTP) being the most frequently used. In the survival category, endpoints were fairly homogeneous with median OS used frequently across the tumor types. OS, in the majority of cases, median OS specifically, was the only “survival” endpoint reported for malignant melanoma and RCC, accounting for 82% and 83% of the “survival” endpoints reported in clinical trial review papers and HTA reports, respectively. Endpoints relating to recurrence and/or relapse were not found in HTA reports but were included in clinical trial review papers. In the review papers, local or overall recurrence rates were the most commonly used.

When comparing the endpoints reported for the different tumor types, there were only minor differences (Figure 2). Survival- and response-related endpoints were more frequent in malignant melanoma, while disease progression and quality-of-life endpoints were more common in RCC.

Several methodological papers have provided recommendations for endpoints that should be considered as part of clinical trial design for malignant melanoma [17], NSCLC [18, 19], and RCC [20, 21] (summarized in Table 3). The recommendations made in these papers largely align with the results from the current literature.

## 4. Different Therapeutic Approaches May Require Different Clinical Value Metrics

Many of the frequently used endpoints were developed primarily to evaluate the clinical activity of standard therapeutic modalities, such as cytotoxic chemotherapy, small-molecule enzyme inhibitors, and radiation. Because of their mechanisms of action, these approaches often have immediate effects that may initially reduce tumor size, but, subsequently, the disease often progresses over time; for patients with advanced disease, they are not generally considered curative. In view of these potential clinical effects, median OS, PFS, and response rate are, in general, appropriate and accurate endpoints for these types of therapies [22].

The evaluation of new therapies with different mechanisms of action may require different or additional metrics to assess and describe value. For example, as discussed earlier, some data suggest that active immunotherapies have the potential for long-term survival, likely achieved through the restoration of durable antitumor immune responses, without necessarily being accompanied by rapid tumor shrinkage [3, 9, 10, 23, 24]. Therefore, different endpoints may be more appropriate for these therapies [17, 22].

### 4.1. Survival-Related Endpoints

OS is considered the gold standard for efficacy in solid tumor oncology clinical trials, and median OS is often quoted as the primary or secondary endpoint of interest. Median OS is often used because it allows survival to be estimated before all patients have experienced an event, that is, death, thereby allowing timely reporting of outcomes.

However, median OS may not be the best endpoint for therapies with potential for long-term benefit [17, 22, 25]. Consider the hypothetical survival curve with a therapy that results in long-term survival in a small proportion of patients versus one with a cytotoxic or targeted therapy that causes an initial, rapid reduction in tumor volume but also no or low prolonged benefit (Figure 3). Median OS is calculated as the point in time after diagnosis or initiation of treatment at which 50% of patients are still alive. However, this assessment may be insufficient for treatments that offer long-term benefit because it does not provide information pertaining to the small proportion of patients who occupy the tail of the survival curve. As such, median OS is considered less suitable for survival curves that are skewed to the right since it does not differentiate the proportion of patients alive or dead after 50% of the patients have died [25]. Furthermore, while median OS provides a measure of when 50% of patients will die, it does not provide a true reflection of the survival time that may be expected from the patients who are alive after the median OS is reached. For example, in a phase 3 trial of ipilimumab (MDX010-020), the median OS of patients who received ipilimumab alone was 10.1 months (*n* = 137) compared with 6.4 months in the control arm (*n* = 136). However, long-term follow-up showed that 13 of the 53 patients (25%) in the ipilimumab group who were randomized ≥3 years before study survival cut-off date survived for 3 years or longer compared with 5 of 50 patients in the control group (10%), an outcome not well reflected by the median OS values [7, 23].

**Figure 1:**
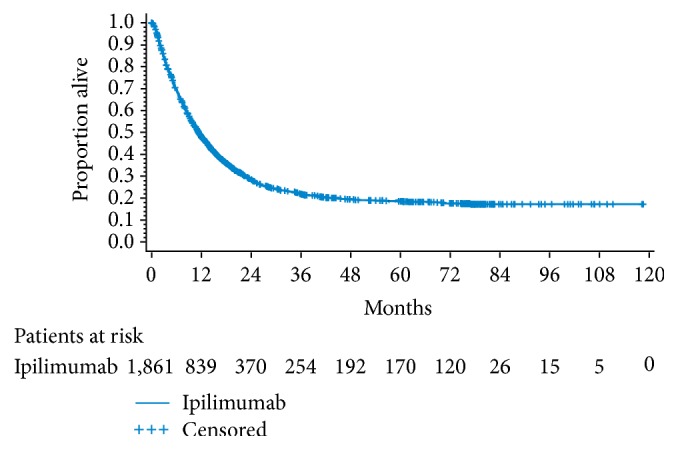
Pooled analysis of 1,861 ipilimumab-treated patients from 12 clinical trials [3]. Median overall survival was 11.4 months (95% CI: 10.7–12.1 months) and 3-year overall survival was 22% (95% CI: 20–24%).

**Figure 2:**
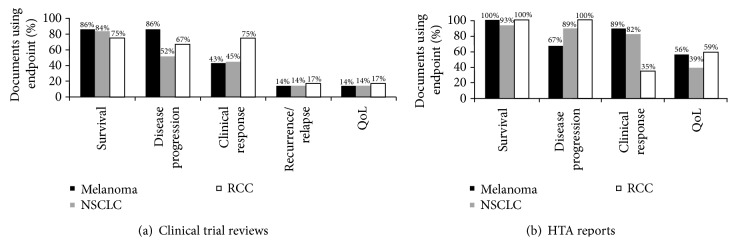
Clinical endpoints used for the different tumor types in (a) clinical trial review papers and (b) health technology assessment reports.

**Figure 3:**
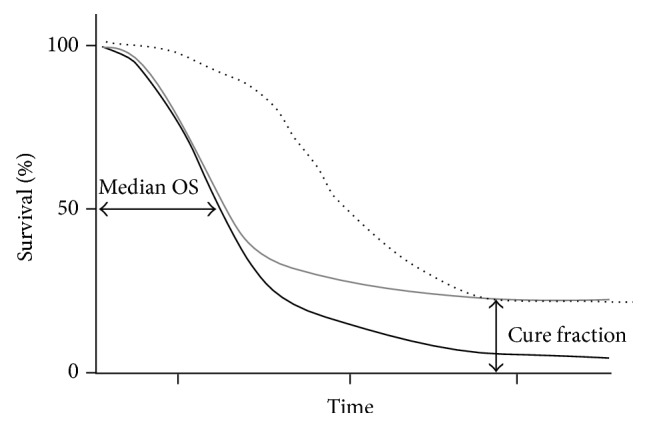
Hypothetical survival curves. The grey line represents an agent (potentially an immunotherapy) that results in long-term benefit in a proportion of patients, the black line represents standard of care (potentially a cytotoxic agent), and the dotted line shows all-cause mortality.

Because of the potential limitations of median OS in describing value of treatments where a proportion of patients experience durable survival, alternative or additional measures of survival may be required. A recent report suggested that combining a robust HR (less than 0.8) with a corresponding improvement in median OS (within a range of 2.5 to 6 months) may define a minimum clinical outcome that could serve as the starting point of a discussion about the medical value of a new treatment [26]. Additionally, other publications have provided examples of how median OS can be supplemented with mean OS as an additional endpoint of interest (as measured by the area under the Kaplan-Meier curve) [23, 25, 27–29]. Other alternatives to median OS are endpoints relating to landmark survival rates, for example at 2, 3, and 5 years after the start of therapy or whichever duration is appropriate to the type of therapy. Although long-term trial follow-up is required, landmark survival at up to 5 years after treatment has been reported with some agents for some solid tumors and hematologic malignancies [7, 8, 30, 31]. In the pooled analysis of ipilimumab-treated patients, the survival rate at 3 years was 22%, and this marked the start of an OS plateau that extended through at least 10 years in some patients (Figure 1). However, these data also reflect the fact that almost 80% of the patients had died within 3 years of treatment initiation [3].

Another potential metric for therapies with potential long-term survival is cure fraction (the proportion of patients who survive and no longer experience the excess mortality rate of the disease) [12]. As discussed above, a “cure” is defined here as a patient population that has the same chance (based on HR) of dying as a member of the general population, depending on the mechanism of action of the agent and the cancer type. This can be achieved through a relatively short course treatment or continual therapy allowing the cancer to be treated as a chronic disease. Standard survival analysis methods, like the Cox proportional hazards model, provide no direct estimate of the cure fraction. However, it may be appropriate to use cure-fraction models for survival data, if a proportion of patients may be cured by a treatment [32]. Such models have been explored for glioblastoma, colon cancer, and breast cancer [33–35].

Against the background of these alternative survival endpoints, reports on the approval of new oncologic drugs and associated documentation show increasing acceptance by HTA bodies of survival endpoints other than median OS [21, 36]. For example, in 2012, mean OS was accepted by the National Institute for Health and Care Excellence (NICE) in the United Kingdom as part of the submission for ipilimumab for previously treated advanced (unresectable or metastatic) melanoma [37]. Furthermore, some HTA bodies are consistently using mean OS in economic models and these models can relatively accurately predict the proportion of patients alive at specific time points. Typically, mean OS is calculated as the area under the Kaplan Meier curve (as an adjusted mean) [25].

### 4.2. Progression-Related Endpoints

Recently, the United States FDA and the European Medicines Agency (EMA) have shown willingness to accept other endpoints as surrogates for OS benefit; PFS and DFS are frequently used, as reflected by the literature review. These are attractive alternatives to median OS since they can be determined earlier, are less influenced by competing causes of death, and are not influenced by second-line therapies [22, 38]. Between July 2005 and December 2007, the FDA approved 44 new products; of these, four used DFS, three used PFS and OS, 11 used PFS or TTP, and 10 used ORR [1]. In Europe, the EMA updated their clinical trial guidelines in 2012 and currently accepts PFS and DFS as primary endpoints in oncology trials [36]. However, a correlation between PFS and OS has only been reported for a limited number of tumor types [22, 38].

PFS and DFS are appropriate for assessing the activity of agents likely to elicit rapid control of tumor growth, but they may be less suitable for therapies where tumor control may develop over time. In some patients receiving immunotherapies, their disease may apparently progress in size (as assessed using standard RECIST or WHO criteria) before there is evidence of disease stabilization, or there may be progression in some lesions while others regress. Some patients may have prolonged disease stabilization, which may evolve over time to become a partial or even complete response, despite sometimes being preceded by progressive disease. These types of response patterns may reflect the time it can take the immunotherapy to modulate the immune system and achieve clinically effective tumor control, and how the immune system responds to an evolving tumor [39, 40]. However, this is not the case for all immunotherapies. Other data show that some patients treated with immunotherapies can have rapid, more conventional types of response [24].

Endpoints such as DFS or PFS may underestimate the activity of novel therapies if associated with prolonged stable disease or unconventional responses, even though these responses may translate into a prolonged survival benefit [39, 40]. As an example, in a phase 3 trial of sipuleucel-T, a therapeutic cancer vaccine, in patients with metastatic castration-resistant prostate cancer, treatment resulted in an improvement in median overall survival (25.8 months with sipuleucel-T versus 21.7 months with placebo; HR for death in the sipuleucel-T group, 0.78; 95% confidence interval [CI], and 0.62–0.98; *P* = 0.03). However, the median PFS was similar in the sipuleucel-T and placebo groups (3.7 months versus 3.6 months) [41]. Similarly, in a randomized phase 2 trial with another investigational cancer vaccine, PROSTVAC-VF, the vaccine improved median OS but not PFS [42].

### 4.3. Response-Related Endpoints

Response rate is another frequently used surrogate endpoint and has been the basis for drug approvals [1]. However, response-related endpoints may not be appropriate for treatments that do not always act by eliciting rapid shrinkages in tumor volume [22]. Data for immunotherapies show that although some patients attain a durable survival benefit, response rates may be lower than expected. This is because response rate, as assessed using standard criteria, may not be a good surrogate for OS if therapies have the potential for prolonged stable disease or other unconventional responses [39, 40]. In a phase 3 trial with ipilimumab in patients with advanced melanoma, the 2-year survival rate was 23.5% in patients who received ipilimumab alone, while the objective response rate was only 10.9% [23].

In addition to endpoints directly related to efficacy, health-related quality of life (HRQoL) is increasingly being recognized as an important endpoint in oncology clinical trials. In patients with advanced disease and a limited life expectancy, depending on the therapy being evaluated, survival alone may not be an appropriate endpoint and improving or maintaining HRQoL becomes a priority. The two most commonly used questionnaires for determining HRQoL in cancer patients are the European Organisation for the Research and Treatment of Cancer Quality of Life Questionnaire (EORTC-QLQ)-C39 and the Functional Assessment of Cancer Therapy-General (FACT-G) [43]. EMA clinical trial guidelines were updated in 2012 and suggest that HRQoL may be an informative endpoint, especially in the palliative setting [36].

## 5. Conclusions

With the emergence of immunotherapy and other new modalities, we are reassessing our expectations for the treatment of patients with advanced solid tumors. For certain patient populations, we are starting to see, either in practice or clinical trials, therapies with the potential for long-term survival and even cure. It is important to consider whether the commonly used endpoints, that is, median OS, PFS, DFS, and response rate, are appropriate to describe value, especially when the new agents have mechanisms of action that may translate into different clinical effects. On reviewing the activity observed with different active immunotherapies in clinical trials, it is clear that* median* OS, PFS, DFS, and response rate may not adequately capture the potential outcomes with these therapies and additional metrics may be needed.

More appropriate or additional metrics for therapies with the potential for long-term survival may include* mean* OS, cure fraction, and OS rate at landmark time points. Encouragingly, recent approvals suggest that agencies like the FDA and EMA are willing to accept additional metrics if they better characterize the agent's activity, although HTA bodies, such as NICE and the Institute for Quality and Efficiency in Health Care (IQWiG), may require different or additional metrics for reimbursement assessment. Furthermore, as the results of the literature review show, cure is a defined and utilized concept for cancers where potentially curative treatments are available. However, the description of cure is relatively heterogeneous and differs depending on the person or group. Because most advanced solid tumors are currently considered incurable with available therapies, the literature review showed that endpoints commonly used in clinical trials of malignant melanoma, NSCLC, and RCC are not well aligned with how cure is described in other cancers. In view of this, if we continue to see new therapies being developed with the potential for long-term survival, then efforts should be made to use the appropriate endpoints and the related set of value metrics that best describe the clinical and other outcomes of these new treatments.

## Supplementary Material

Supplementary Material 1 provides an overview of the objectives, methodology, outcomes and limitations, and results of the literature review conducted to define how various stakeholders describe cure in oncology.
Supplementary Material 2 provides an overview of the objectives and outcomes and limitations of the literature review conducted to identify the clinical endpoints currently used in malignant melanoma, non-small cell lung cancer, and renal cell carcinoma trials.

## Figures and Tables

**Table 1:** Endpoints identified in clinical trial reviews.

Category^a^	Endpoint reported (% of category) (*N* = 266)			
	Included in >1 cancer type	Malignant melanoma only	NSCLC only	RCC only
Clinical response (*n* = 79)	Complication rate (26%) Response rate (23%) Local control/local tumor control (9%) Absolute benefit (8%) Disease control rate (3%) Complete response (1%)	Partial response (3%)	Time to progression (8%) Objective response rate (4%) Tumor response (3%) Symptom improvement (3%) 3-year freedom from local progression (1%) Length of hospital stay (1%) Radiological improvement (1%) Treatment failure rate (1%) Tumor progression (1%) Postoperative complication rate (1%)	Positive margin rate (1%) Surgery success rate (1%)

Disease progression (*n* = 66)	Progression-free survival (36%) Disease-free survival (32%) Recurrence-free survival (23%)	Distant metastasis-free survival (2%) Locoregional PFS (2%)	Asymptomatic survival (2%) Quality-adjusted PFS (2%)	

Survival (*n* = 87)	OS^b^ (82%)		Drug related deaths (6%) 5-year survival (3%) Cause-specific mortality (3%) 1-year survival (2%) Treatment mortality/morbidity (2%) Perioperative morbidity (1%)	

Recurrence/relapse (*n* = 21)	Local recurrence rate (38%) Recurrence rate (overall) (14%)		Relapse (14%) Asymptomatic recurrence (5%) Distant recurrence (5%) Remission (5%) Symptom-free period (5%) Systemic recurrence (5%) Time to recurrence (5%) Time to relapse (5%)	

QoL (*n* = 13)	Health-related QoL (8%)	QoL (92%)		

^a^Review papers may include more than one endpoint category.

^b^Included review papers of clinical trials that did not consistently report whether the articles they referenced used median or mean OS. When mentioned, the majority of included trial review papers referred to median OS.

**Table 2:** Endpoints identified in health technology assessment reports.

Category^a^	Endpoint reported (% of category) *N* = 232			
	Included in >1 cancer type	Malignant melanoma only	NSCLC only	RCC only
Clinical response (*n* = 79)	Complete response (11%) Duration of response (13%) Objective response rate (13%) Overall response rate (12%) Partial response (10%) Objective tumor response (8%) Disease control rate (4%) Stable disease (4%) Time to response (3%) Response rates (1%)	Disease progression (1%) Near complete response (1%)	Tumor response (12%) Best tumor response (1%) Physical functioning (1%) Symptomatic improvement (1%) Time to tumor progression (1%) Time to worsening of patient reported outcomes (1%) Tolerance (1%)	

Disease progression (*n* = 65)	Progression-free survival (72%) Time to progression/progressive disease (15%)		Time to treatment failure (8%) Time to worsening of symptoms (3%)	Time to first event (2%)

Survival (*n* = 63)	Median OS (83%) 1-year survival (13%) 2-year survival (5%)			

QoL (*n* = 25)	QoL (68%) Health-related QoL (20%)		Disease-specific questionnaire (4%) Lung cancer symptom scale (4%)	Patient reported outcomes (4%)

^a^HTA reports may include more than one endpoint category.

**Table 3:** Recommended clinical endpoints for trials in patients with malignant melanoma, non-small cell lung cancer, or renal cell carcinoma.

Malignant melanoma [17]	NSCLC [18, 19]	RCC [20, 21]
Overall survival^a^	Disease stability	Median overall survival

Progression-free survival	Growth modulation index	Median progression-free survival

Quality of life	Median overall survival	Response rate

	Median progression-free survival	

	Time to progression	

^a^The article did not specify median or mean OS.
