# Supplementary material for: Which Metrics Are Appropriate to Describe the Value of New Cancer Therapies?
Source: Biomed Res Int. 2015 Jun 16;2015:865101. doi: 10.1155/2015/865101 (PMC4486603; doi:10.1155/2015/865101)
Supplement: Supplementary file 1 — Supplementary Material 1 provides an overview of the objectives, methodology, outcomes and limitations, and results of the literature review conducted to define how various stakeholders describe cure in oncology. Supplementary Material 2 provides an overview of the objectives and outcomes and limitations of the literature review conducted to identify the clinical endpoints currently used in malignant melanoma, non-small cell lung cancer, and renal cell carcinoma trials. [file 865101.f1.pdf]

Supplementary Material 1: Literature Review of Cancer Cure Definitions

Objective

To identify how key stakeholders including health technology assessment (HTA) bodies, clinicians, and patients currently define "cancer cure."

Methodology

The literature search focused on four stakeholder groups including patient advocacy groups, clinicians, academics, and HTA bodies. This phase included literature from four sources: journal articles, conference and congress proceedings, HTA reports, and selected websites. Journal articles discussing cure or cure models were identified via a MEDLINE® and Embase® literature database search. To verify the appropriate choice and use of search terms, the search was validated against two articles (Lakdawalla et al. 2012; Othus et al. 2012). Searches were restricted to English language papers concerning humans and published between January 1st, 2007 and November 14th, 2012 (data cut-off point). A second MEDLINE® and Embase® search was conducted to identify treatment guidelines concerning malignant melanoma, RCC, and NSCLC. The second search was also restricted to English language articles published between January 1st, 2007 and November 14th, 2012. The literature database search strategy was designed pragmatically to prioritize the identification of key information in a very large literature base. Furthermore, the search strategy included keywords and medical subject heading terms.

In addition, a hand search identified relevant abstracts published between 2010 and 2012 from the American Society of Clinical Oncology (ASCO), European Society for Medical Oncology (ESMO), European Cancer Organisation (ECCO), and the American Society of Hematology (ASH) conferences. HTA reports specific to melanoma, NSCLC, and RCC were identified via HTA websites from England/Wales, Scotland, France, Canada, and Australia. The website literature search included patient/patient interest organizations and professional groups' websites. Patient groups were identified as listed on the European Organisation for Research and Treatment of Cancer (EORTC) website (http://www.eortc.org), and via links on the websites of groups listed by the EORTC. Only websites for groups located in the US, UK, Germany, France, Italy, and Spain, and containing information in English, were included. The websites were searched for definitions of "cancer cure" using the website's search engine and by navigating links to relevant documents.

No predefined restrictions on study design or patient population were applied. However, included data sources were expected to discuss the definition of cure, apply a definition of cure, or highlight the shortcomings and/or limitations of one or more cure definitions and measurements.

Journal articles identified in the literature searches were screened and prioritized on the basis of relevance to the study question (in the first instance) and how recently they were published or made available (in the second instance). The screening process is shown in **Supplementary Figure 1**. Journal article screening was undertaken by one screener in an iterative process, with the inclusion criteria becoming gradually more restrictive until the most valuable information sources were identified. Initially, search results were screened by title and abstract (first screen). Journal articles deemed potentially most relevant were obtained as full text documents. Full-text sources were assessed for further inclusion (second screen). Each citation was screened and extracted by one reviewer. The first and second screens as well as data extractions underwent a quality control check by a second independent reviewer. Websites, conference abstracts, and HTA reports only underwent a full-text screen. This was identical to the second screen step used for journal articles.

Outcomes and Limitations

The following data were targeted:

- Cure definitions regardless of cancer type
- Discussion around the definition and ranking of cancer cure

- Data indicating consensus opinions on the most valuable treatment endpoints to measure cure

- Discussion around the limitations of current treatment endpoints in measuring cure

- The impact and balance of maximizing treatment efficacy compared to the value of minimizing adverse events. Particular attention was paid to definitions and concepts that were mentioned across several sources, and which national HTA agencies focused on during their decision-making processes.

Cure definitions obtained from conference abstracts and journal articles were stratified according to the affiliation of the first author (academic or clinician). All extracted definitions were divided into seven categories based on similarity. These included disease progression, eradication of cancerous cells, survival, reduced relapse rates, disease progression and increased survival, eradication of cancerous cells and absence of recurrence, and miscellaneous. The ‘disease progression’ category included definitions, where an absence of disease progression was the primary criterion for defining cure. The ‘eradication of cancerous cells’ category included statements, which defined cure as the successful removal of cancerous cells. The ‘survival’ category included definitions, where an increase in survival was the main criterion for defining cure. The ‘reduced relapse rates’ included definitions, where cure was defined as the absence of relapse. Furthermore, the two categories, ‘disease progression and increased survival’ and ‘eradication of cancerous cells and absence of recurrence,’ included definitions covering more than one of the previously stated categories (i.e., a definition that referred to both an absence of disease progression and a positive effect on survival, or successful eradication of cancerous cells and lack of recurrence). Finally, the ‘miscellaneous’ category included a single statement that did not fit into any of the other categories and which provided information on what did not constitute a cure.

Following an assessment of the data, documents were stratified according to publication year to give insight into possible time trends. During this step, definitions from patients and non-profit organizations were excluded as they had been obtained from websites, where the exact publication date could not be determined. Definitions from HTA documents were also excluded due to the small sample size (*n*=4).

While this review provided insight into expectations regarding curative cancer, certain methodological limitations must be noted. First, the database searches were limited by publication date and language. Similarly, the conference abstract search was limited to pre-determined conferences, and, as such, neither search was exhaustive. A second limitation relates to the HTA searches, which were restricted to the UK, France, Canada, and Australia. These four countries were chosen because of the high quality of and open access to their HTA reports; however, it was beyond the scope of the current review to determine the extent to which these payer-relevant definitions of "cure" and emphasis on different clinical endpoints apply to other countries. Third, it was not possible to use search facets to identify information from web-based literature sources and these were, therefore, hand searched. The risk for a potential bias or inconsistent identification of web-based literature data was, however, minimized by using the predetermined search strategies. Fourth, it should be noted that the time trend analysis was conducted on a relatively small sample and during a short time window. Whilst showing short-term trends, these results may, therefore, not be appropriate for drawing conclusions on long-term changes in stakeholders’ expectations for curative treatments.

Results

The literature review identified 3,932 documents, of which 169 were included following screening. The final sample included journal articles (*n*=83), HTA documents (*n*=7), conference abstracts (*n*=56), and documents from patients/patient advocacy organizations (*n*=18) and health care professionals’ (*n*=5) websites. Cure definitions were extracted from all included documents and categorized into seven categories (**Supplementary Table 1**).

The literature review identified a range of "cure" definitions, which differed both within and between the stakeholder groups. Health-care professionals, i.e., clinicians, and academics most commonly defined "cure" using definitions that fell into the survival (35% and 39%,

respectively) or disease progression categories (28% and 39%, respectively) (**Supplementary Figure 2**). Among clinicians, the most commonly used definition in the 'disease progression' category was relapse-free survival (RFS) (33% of responses in the 'disease progression' category). Similarly, academics, who defined 'cure' using definitions that fell into the 'disease progression category,' also most commonly referred to RFS (43% of responses in the 'disease progression' category). While using similar definitions of 'cure,' the two stakeholder groups differed in their methodology for measuring each endpoint. Whereas healthcare professionals predominantly measured RFS in terms of 'x' years of RFS, academics more commonly used statistical models that determined the proportion of patients who would not experience a recurrence of the disease.

Unlike healthcare professionals and academics, the majority of patients and patient-advocacy groups (72%) defined "cure" using definitions that broadly fell into the 'eradication of cancerous cells' category. When defining "cure" in these terms, it was either with or without mentioning a requirement for an absence of recurrence (39% and 33%, respectively). There was a similar trend among payers, where 57% of the seven documents defined "cure" as the successful eradication of cancer cells. However, payers used more homogenous definitions with definitions falling into the 'eradication of cancerous cells' category exclusively relating to the surgical removal of cancerous cells. Patients and patient-advocacy organizations used multiple and less quantifiable definitions.

When measuring 'cure' in the context of increased survival, patient and patient-advocacy groups noted an average follow-up period of 5 years. This aligned with the views of healthcare professionals, who reported an average follow-up period of 5 years and 11 months. However, there were differences in the methods, which different stakeholders used to measure survival. The majority of healthcare professionals and patients and non-profit organizations' definitions of 'cure,' which fell into the 'survival' category, measured survival in terms of 'x' years of survivorship. Conversely, academics' 'cure' definitions that fell into the 'survival' category more commonly measured survival using statistical models to predict cure fraction.

References

D. N. Lakdawalla, J. A. Romley, Y. Sanchez, et al., "How cancer patients value hope and the implications for cost-effectiveness assessments of high-cost cancer therapies," *Health Aff (Millwood)* vol. 31, no. 4, pp. 676-682, 2012.

M. Othus, B. Barlogie, M. L. Leblanc, J. J. Crowley, "Cure models as a useful statistical tool for analyzing survival," *Clin Cancer Res* vol. 18, no. 14, pp. 3731-3736, 2012.

Supplementary Table 1: Categories of cancer cure definitions by group

Category	Clinicians	Academics	Patients and patient advocacy organizations	Payers
Disease progression	Complete remission<br>Disappearance of clinical evidence of disease<br>DFS<br>FFS<br>Functional cure<br>PFS<br>RFS<br>Time to treatment failure	Disease-free survival<br>Failure-free survival<br>Long-term remission<br>RFS<br>Therapy discontinuation without relapse		"Disappearance of all visible disease"<br>DFS<br>"Substantial restoration of health (30-year perspective)"
Disease progression & increased survival	Complete remission and improved survival<br>Improved survival without relapse<br>OS and DFS<br>OS, DFS, and EFS<br>Permanent survival phase (i.e., low likelihood or return of primary disease)<br>OS and PFS<br>OS and time to progression			
Eradication of cancerous cells	Eradication of leukemic stem cells<br>Surgical removal of cancerous cells<br>Radiation therapy	Eradication of leukemic stem cells (method not specified)<br>Pharmacological eradication of cancerous cells<br>Surgical eradication of cancerous cells	Disrupting cancer cells ability to divide and reproduce<br>"Kill cancer cells"<br>Local tumor cure<br>Radiotherapy<br>Surgical removal of cancerous cells<br>"Take away all the cancer"	Surgical removal of cancerous cells
Eradication of cancerous cells & absence of recurrence			'x' years survival without recurrence<br>Removal of cancerous cells without recurrence	
Survival	2-year OS<br>3-year OS<br>5- year OS<br>10-year OS<br>20-year OS<br>Cure model<br>OS (non-specific)<br>Patient dies from non-cancer related cause	5-year survival<br>Cure fraction – same survival as general population<br>"Long-term survivors"	5-year OS<br>"[cure is] living a long life"<br>Mortality rates aligning with the general populations<br>OS (non-specific)	
Miscellaneous		"Complete molecular response does not equal cure"		
Reduced relapse rates			Reduced relapse rates	

DFS, disease-free survival; EFS, event-free survival; FFS, failure-free survival; OS, overall survival; PFS, progression-free survival; RFS, relapse-free survival.

Supplementary Figure 1: Methodology used to identify and extract data from journal articles (left) and websites, conference abstracts, and HTA reports (right)

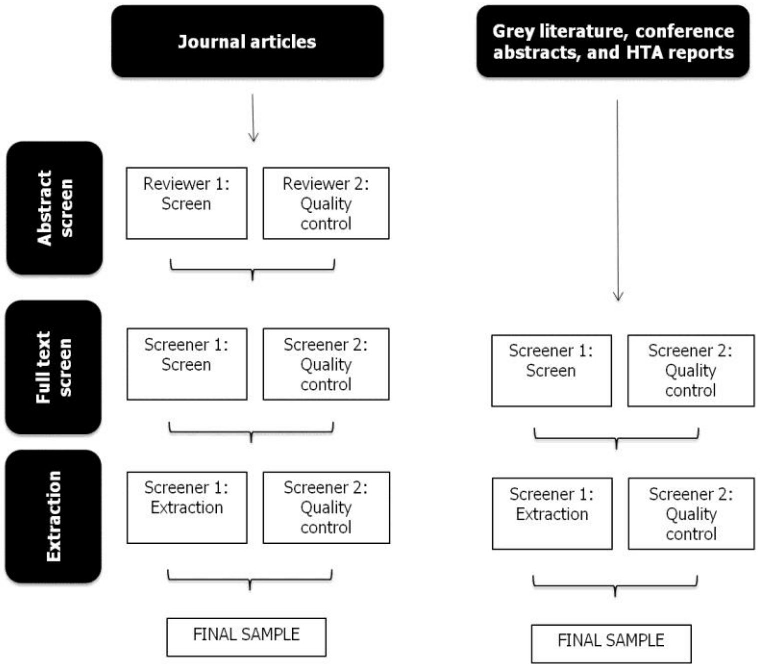

Supplementary Figure 2: Definitions of ‘cure’ obtained from documents published by (A) health care professionals, (B) academics, (C) patients and patient advocacy groups (non-profit organizations), and (D) HTA agencies (referred to as payers).

The bar chart shows the percentage of documents within each group using the respective definitions.

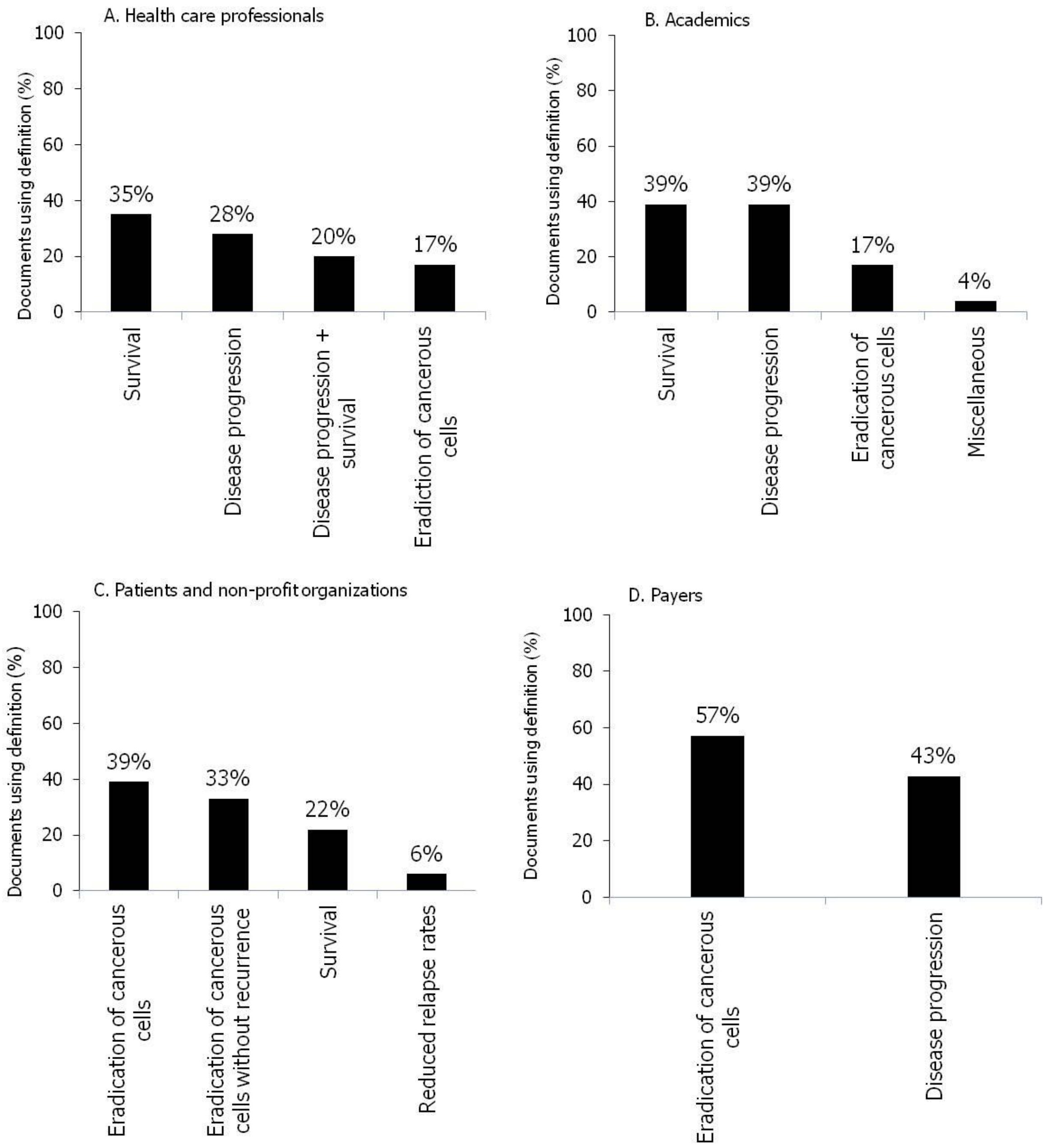

Supplementary Material 2: Literature Review of Cancer Treatment Endpoints

Objective

To identify the clinical endpoints currently used in malignant melanoma, non-small cell lung cancer (NSCLC), and renal cell carcinoma (RCC) trials.

Methodology

The literature search included published systematic reviews of clinical trials and health technology assessment (HTA) reports. Articles identified from the cure definition search (Supplementary Material 1) referring to ‘cure’ in conjunction with endpoints for malignant melanoma, RCC, and NSCLC were also included in this review. In addition, a MEDLINE® and Embase® database search was conducted to identify reviews of clinical trials focusing on malignant melanoma, RCC, or NSCLC. Searches were restricted to English language articles published between January 1st, 2007 and December 31st, 2012. The literature database search strategy was designed pragmatically to prioritize the identification of key information in a very large literature base. Furthermore, the search strategy included keywords and medical subject heading terms. No predefined restrictions on study design or patient population were applied. However, articles discussing endpoints used in conjunction with definitions of ‘cure’ or reporting endpoints commonly used in relevant oncology clinical trials were prioritized for inclusion.

Journal articles identified in the literature search were screened and prioritized on the basis of relevance to the study question (in the first instance) and how recently they were published or made available (in the second instance). The screening process is shown in **Supplementary Material 1, Figure 1**. Journal article screening was undertaken by one screener in an iterative process, with the inclusion criteria becoming gradually more restrictive until the most valuable information sources were identified. Initially, search results were screened by title and abstract (first screen). Journal articles deemed potentially most relevant were obtained as full text documents. Full-text sources were assessed for further inclusion (second screen). Each citation was screened and extracted by one reviewer. The first and second screens as well as data extractions underwent a quality control check by a second independent reviewer. HTA reports only underwent a full-text screen.

Outcomes and Limitations

Reviews of clinical trials and HTA reports, focusing on malignant melanoma, NSCLC, or RCC, were screened, and the clinical endpoints reported in these documents were extracted. A list of cited clinical endpoints and the frequency with which they were used was generated. Only endpoints directly referring to clinically measured outcomes were included. Further, point estimates such as hazard ratios or relative risks were not extracted as these relate to the analysis of data post-collection. Moreover, when extracting data from HTA reports, only final appraisal documents were considered.

The extracted endpoints were divided into five categories, depending on similarity. These categories were clinical response, disease progression, survival, recurrence/relapse, and Quality of Life (QoL). The ‘clinical response’ category included endpoints measuring the short-term response to treatment (e.g., tumor response) and tumor-specific responses (e.g., tumor progression). The ‘disease progression’ category included endpoints measuring long-term outcomes associated with an absence of cancer (e.g., disease-free survival). The ‘survival’ category included measurements of survival that did not take other aspects of disease, such as absence of symptoms, into account (e.g., 5-year survival). The ‘recurrence/relapse’ category included endpoints measuring the absence of relapse or time to relapses (e.g., recurrence rate). Finally, the ‘QoL’ category included endpoints measuring patients’ quality of life.

It should be noted that the search only included published reviews of clinical trials and HTA reports, and was not designed to retrieve an exhaustive list of clinical trial endpoints, but rather to identify the most frequently used clinical outcomes in malignant melanoma, NSCLC, and RCC. Therefore, collected data do not exclude the possibility that less commonly used

clinical outcome measures may relate to the definitions of ‘cure,’ identified in the first literature search.
